# An Ethylene-Protected Achilles’ Heel of Etiolated Seedlings for Arthropod Deterrence

**DOI:** 10.3389/fpls.2016.01246

**Published:** 2016-08-30

**Authors:** Edouard Boex-Fontvieille, Sachin Rustgi, Diter von Wettstein, Stephan Pollmann, Steffen Reinbothe, Christiane Reinbothe

**Affiliations:** ^1^Laboratoire de Génétique Moléculaire des Plantes and Biologie Environnementale et Systémique, Université Grenoble-Alpes – Laboratoire de Bioénergétique Fondamentale et Appliquée Grenoble, France; ^2^Department of Agricultural and Environmental Sciences–Pee Dee Research and Education Center, Clemson University, Florence SC, USA; ^3^Department of Crop and Soil Sciences – Center for Reproductive Biology, School of Molecular Biosciences, Washington State University, Pullman WA, USA; ^4^Centro de Biotecnología y Genómica de Plantas, Univerdidad Politécnica de Madrid – Instituto Nacional de Investigación y Tecnología Agraria y Alimentación Madrid, Spain

**Keywords:** skotomorphogenesis, apical hook, *Arabidopsis thaliana*, protease inhibitor action, herbivore deterrence

## Abstract

A small family of Kunitz protease inhibitors exists in *Arabidopsis thaliana*, a member of which (encoded by At1g72290) accomplishes highly specific roles during plant development. *Arabidopsis* Kunitz-protease inhibitor 1 (Kunitz-PI;1), as we dubbed this protein here, is operative as cysteine PI. Activity measurements revealed that despite the presence of the conserved Kunitz-motif the bacterially expressed Kunitz-PI;1 was unable to inhibit serine proteases such as trypsin and chymotrypsin, but very efficiently inhibited the cysteine protease RESPONSIVE TO DESICCATION 21. Western blotting and cytolocalization studies using mono-specific antibodies recalled Kunitz-PI;1 protein expression in flowers, young siliques and etiolated seedlings. In dark-grown seedlings, maximum *Kunitz-PI;1* promoter activity was detected in the apical hook region and apical parts of the hypocotyls. Immunolocalization confirmed Kunitz-PI;1 expression in these organs and tissues. No transmitting tract (NTT) and HECATE 1 (HEC1), two transcription factors previously implicated in the formation of the female reproductive tract in flowers of *Arabidopsis*, were identified to regulate Kunitz-PI;1 expression in the dark and during greening, with NTT acting negatively and HEC1 acting positively. Laboratory feeding experiments with isopod crustaceans such as *Porcellio scaber* (woodlouse) and *Armadillidium vulgare* (pillbug) pinpointed the apical hook as ethylene-protected Achilles’ heel of etiolated seedlings. Because exogenous application of the ethylene precursor 1-aminocyclopropane-1-carboxylic acid (ACC) and mechanical stress (wounding) strongly up-regulated HEC1-dependent *Kunitz-PI;1* gene expression, our results identify a new circuit controlling herbivore deterrence of etiolated plants in which Kunitz-PI;1 is involved.

## Introduction

When seeds germinate underneath the soil or fallen leaves, they pass through a developmental program known as skotomorphogenesis (von Wettstein et al., 1995). Dark-grown (etiolated) seedlings establish an elongated hypocotyl (epicotyl) terminating in an apical hook with closely apposed, unexpanded cotyledons. This specific morphology and especially the presence of the apical hook enables etiolated seedlings to grow through the soil without mechanical damage (Darwin, 1896). When dark-grown seedlings break through the soil, they switch to another developmental program, that is, photomorphogenesis, during which hypocotyl growth is arrested and unfolding of the cotyledons and greening proceed. Mechanisms have been identified that maintain the skotomorphogenetic program in the dark and trigger photomorphogenesis in the light.

Several phytohormones comprising brassinosteroids, auxins, gibberellins, jasmonic acid, and ethylene regulate skotomorphogenetic growth (Alabadí et al., 2004; Alabadí and Blázquez, 2009; Zhong et al., 2009; Li et al., 2012; Li and He, 2013; Hsieh and Okamoto, 2014). For example, pioneering work performed on pea epicotyls revealed that increased mechanical impedance caused enhanced rates of ethylene biosynthesis (Harpham et al., 1991). As a consequence, an increased radial expansion and decreased elongation of the epicotyl were observed (Goeschl et al., 1966). Ethylene production was confined to the epicotyl hook and plumule and shown to decrease upon illumination with red light (Goeschl et al., 1967). Ethylene was also demonstrated to be necessary for the formation and maintenance of the apical hook in etiolated seedlings of *Arabidopsis thaliana* (Raz and Ecker, 1999).

Analysis of the cell wall proteome corresponding to different stages of hypocotyl elongation of etiolated seedlings revealed a great dynamics in cell wall protein composition in *Arabidopsis* (Irshad et al., 2008). Among the identified proteins were aspartate, cysteine, and serine proteases as well PIs of the Kunitz family (Irshad et al., 2008). Both ethylene and proteases are normally implicated in controlling PCD in a vast range of physiological contexts, including the HR to pathogen attack, tracheary-element differentiation, and senescence. For example, some fungal elicitors were shown to induce ethylene biosynthesis and PCD in tobacco leaves (Anderson et al., 1982). It was observed that treatment with phenylmethanesulfonyl fluoride (PMSF) and soybean trypsin inhibitor (two serine PIs), but not pepstatin A (a carboxyl PI) abrogated this response (Anderson et al., 1982). Other studies have implemented ethylene and protease action in PCD during the HR to pathogen attack (Beers et al., 2000), oxidative stress (Solomon et al., 1999), leaf senescence (Chen et al., 2002), and flower petal senescence (Jones et al., 1995). The fungal elicitor ethylene-inducing xylanase (EIX) was shown to elicit ethylene biosynthesis in tomato and tobacco leaves through induction of ACC synthase gene expression. Evidence was obtained for a role of a cysteine protease in controlling ACC synthase expression (Matarasso et al., 2005). The protease specifically binds to a *cis*-element of the ACC synthase gene and acts as a transcription factor. Last but not least, plant responses to ethylene are mediated by SCF(EBF1/EBF2)-dependent proteolysis of EIN3 transcription factor (Guo and Ecker, 2003; Christians et al., 2008; An et al., 2010), highlighting the role of proteolysis in ethylene signal transduction.

With regard to skotomorphogenesis, it is somewhat unexpected to see aspartate, cysteine and serine proteases accumulating. However, their location in cell walls is rather suggestive of a direct defense function against biotic foes. On the other hand, endogenous PIs such as Kunitz-protease inhibitors could regulate their activity. Because Kunitz-protease inhibitors were also identified in the cell wall proteome of the elongating parts of dark-grown chickpea (*Cicer arietinum*), a role in growth regulation was proposed (Jiménez et al., 2007; Hernández-Nistal et al., 2009). Here, we report on a Kunitz-protease inhibitor that accumulates in the apical hook of etiolated *Arabidopsis* seedlings and is part of a mechanism of arthropod deterrence through which young-born seedlings are protected against herbivory during greening (Boex-Fontvieille et al., 2015a). Expression studies of this novel Kunitz-PI, termed Kunitz-PI;1, identified a new regulatory circuit that comprises ethylene, auxin, and the transcription factors NTT and HEC1, previously implicated in female reproductive tract development in flowers of *A. thaliana* (Crawford et al., 2007; Gremski et al., 2007). Together, our results provide new insights into the mechanisms that govern skotomorphogenesis in the model plant *Arabidopsis*.

## Materials and Methods

### Plant Material

The following *A. thaliana* genotypes were used in this study: Columbia (Col-0; referred to as wild-type, WT), SALK_009681 (renamed to *Kunitz-PI;1*) that carries a T-DNA insertion in the At1g72290 gene encoding the Kunitz-PI;1 (Boex-Fontvieille et al., 2015a), Kunitz-PI;1 overexpressor (WT transformed with the plasmid pB7WG2 containing the coding frame for Kunitz-PI;1, referred to as *35S::Kunitz-PI;1*; Boex-Fontvieille et al., 2015a), Kunitz-PI;1 promoter-ß-glucuronidase (*pKunitz-PI;1::GUS*) consisting of the promoter region of Kunitz-PI;1 in front of the ß-glucuronidase coding sequence (Boex-Fontvieille et al., 2015a), *ntt-2* (SALK_007406; Alonso et al., 2003; Crawford et al., 2007), *hec1* (GABI-KAT 297B10), *hec3* (SALK_005294, Alonso et al., 2003), and *hec1::hec3* (Alonso et al., 2003; Gremski et al., 2007).

### Growth Conditions

Dark- and light-grown seedlings were obtained from seeds that had been surface-sterilized by imbibition in hypochlorite solution and ethanol. Seeds were plated on petri dishes containing Murashige–Skoog mineral salts (Sigma–Aldrich; 4.3 g/L), MES (0.5 g/L), and agar (10 g/L), pH 5.7, and kept in the dark at 4°C for 48 h. Germination was induced by illumination with white light of 70 μE m^-2^s^-1^ for 3 h. The plates were then either returned to darkness or kept in white light for appropriate periods. Plates to be used for phytohormone tests contained 10 μM IAA, 10 μM ACC, or 100 μM silver nitrate (AgNO_3_). For seed production, seedlings were grown to maturity on soil in a culture room in 16 h light/8 h dark cycles at 70 μM × s^-1^ × cm^-2^.

### Protein Expression and Purification

cDNA encoding the precursor Kunitz-PI;1 protein including the predicted NH_2_-terminal, 23 amino acids signal sequence^1^ was amplified by PCR (Innis et al., 1990) with primers 5′-GGGGACAAGTTTGTACAAAAAAGCAGGCTTCAAGAATCCTTCAGTGATCTCTTTT-3′ and 5′-GGGGACCACTTTGTACAAGAAAGCTGGGTCTCAACCCGGGAAGTATAAGTTGCT-3′. Similarly, cDNA encoding the predicted mature Kunitz-PI;1 protein was amplified with the primers 5′-GGGGACAAGTTTGTACAAAAAAGCAGGCTTCCACGGAAATGAACCGGTG-3′ and 5′-GGGGACCACTTTGTACAAGAAAGCTGGGTCTCAACCCGGGAAGTATAAGTTGCT-3′. The PCR products were cloned into pDONR221 (Plant System Biology, VIB-Ghent University) using Gateway technology (Invitrogen). For allowing protein expression, the cDNA were introduced into pDEST17 (Plant System Biology, VIB-Ghent University) and then used to transform *Escherichia coli*, strain BL21. The NH_2_-terminal (His)_6_-tagged Kunitz-PI;1 precursor or Kunitz-PI;1 mature protein was expressed after induction with arabinose (0.2% [w/v] final concentration) and growth at 37°C for 3 h. Pellets obtained from 0.5 L-batches of the bacterial cultures were lysed in a buffer containing 20 mM NaH_2_PO_4_, pH 7.5, 6 M urea, 20 mM imidazole, 500 mM NaCl and 0.5 mM PMSF and passed through a French Press (Thermo Electron, FA-078A). After centrifugation, the clear lysate was subjected to Ni-NTA agarose chromatography according to the manufactures instructions (Qiagen). Approximately 90–95% pure protein was obtained.

### Determination of Protease Inhibitor Activity

Pilot experiments were carried out as described (Halls et al., 2006; Homaei et al., 2010; Boex-Fontvieille et al., 2015b), using 10^-3^ to 10^-6^ moles of bacterially expressed and purified Kunitz-PI;1 or soybean trypsin inhibitor and a fixed, 10^-6^ molar concentration of papain or trypsin (all chemicals from Sigma–Aldrich). The results shown in **Supplementary Figure S1** refer to a 10-fold molar excess of PI to protease and refer to three independent experiments. In more refined studies, purified Kunitz-PI;1 was mixed with 6 × 10^-6^ moles of trypsin from swine pancreas (Sigma–Aldrich) at molar ratios of either 1:1 or 1:10 in 2.5 mL of reaction buffer containing 50 mM Tris-HCl, pH 7.5, and incubated for 5 min at 25°C. Then, a 2 mL-aliquot was withdrawn and mixed with 100 μL benzoyl-L-arginine ethyl ester (BAEE, 10 mM stock solution). Substrate conversion was followed over a period of 4 min by absorbance measurements at 253 nm in a Cari 100 spectrophotometer. Control incubations contained soybean trypsin inhibitor (3.10^-9^ moles per assay; Sigma–Aldrich).

Reverse zymography was carried out on SDS-polyacrylamide (PAA) gels containing 0.1% (w/v) porcine skin gelatine (Sigma–Aldrich). After electrophoresis, the gels were incubated twice for 30 min in a buffer containing 0.1 M Tris-HCl, pH 7.5, and 2.5% (v/v) Triton X100. The gels were rinsed several times with distilled water and successively incubated in 50 mM Tris-HCl, pH 8.2, supplemented with either trypsin or chymotrypsin at a concentration of 0.1% (v/v), at 4°C for 30 min and at 37°C for 90 min. Thereafter, the gels were briefly rinsed with distilled water and stained with Coomassie brilliant blue.

### Protein Analyses

Either etiolated seedlings or their upper parts comprising the apical hook and the cotyledons were frozen in liquid nitrogen and ground in a mortar. Three-hundred mg of the resulting powder was mixed with 600 μL extraction buffer containing 0.5 M Tris-HCl, pH 8, 5 mM EDTA, 0.1 M NaCl, 10 % (v/v) ß-mercaptoethanol, 2 mM PMSF, and 0.7 M sucrose. After addition of one volume of phenol (equilibrated with 10 mM Tris-HCl, pH 8) the tubes were shaken at room temperature for 20 min. The upper phase obtained after centrifugation at 12,000 rpm in an Eppendorf microcentrifuge, model 5414, was aspirated with a pipette, transferred into a new test tube and mixed with five volumes of ammonium acetate (0.1 M in methanol). Protein was precipitated at -20°C for 2 h and pelleted by centrifugation at 12,000 rpm at 4°C for 5 min. The pellets were washed twice with ammonium acetate and once with 500 μL 80% (v/v) acetone, dried at room temperature, dissolved in 100 μL twofold concentrated SDS sample buffer containing 125 mM Tris-HCl, pH 6.8, 20% (v/v) glycerol, 4% (w/v) SDS, 10% (v/v) ß-mercaptoethanol, and boiled at 96°C for 5 min. Protein from all other types of plant samples was obtained by a quick-step method of boiling plant tissue powders in SDS-sample buffer at 100°C for 1 min. The homogenates were cleared by centrifugation at 8,000 rpm for 1 min and the supernatant transferred to a new test tube. Protein concentration was determined according to Esen (1978). SDS-PAGE was carried out on 12% (w/v) PAA gels containing SDS. After electrophoresis, protein was blotted onto nitrocellulose membranes (0.45 μm, Whatman) according to Towbin et al. (1979). Immunodetection of protein was made using Kunitz-PI;1 antibodies (Boex-Fontvieille et al., 2015a) and alkaline phosphatase-conjugated goat anti-rabbit antibodies (diluted 1:3000) or horseradish peroxidase-conjugated goat anti-rabbit antibodies (diluted 1:5000). Crossreactive bands were detected with 5-bromo-4-chloro-3-indolyl phosphate (BCIP) and nitro blue tetrazolium (NBT) or via enhanced chemiluminescence (ECL Western Blotting Analysis system, Amersham), respectively.

### Immunolocalization

Etiolated seedlings were infiltrated for 10 min in a solution containing 5% (v/v) glacial acetic acid, 3.7% (v/v) formaldehyde and 50% (v/v) ethanol and incubated for 24 h at 4°C. Thereafter, the samples were dehydrated by successive incubations in solutions of increasing ethanol concentrations (70% [v/v], 90% [v/v], and 100% [v/v], 1 h each) and Histoclear (25% [v/v], 50% [v/v], 75% [v/v], and 100% [v/v], 1 h each). Embedding was done in paraffin (Paraplast X-TRA, Tyco Healthcare). Ten-μm tissue-sections were prepared with a microtome (Microtom HM 355S, Zeiss), mounted on glass slides and incubated for 3 h at 45°C. Paraffin was removed by two incubations with Histoclear, for 2 × 10 min each. Rehydration of samples was achieved by successive incubations in solutions of decreasing ethanol concentrations.

For immunolocalization, the samples were first depleted of endogenous alkaline phosphatase activity^2^ Tissue sections were incubated at 95°C for 40 min in buffer consisting of 10 mM citric acid, pH 6, and 0.05% (v/v) Tween 20, then cooled to room temperature, and finally incubated in a solution containing 0.05% (v/v) Tween 20 and 5% (w/v) low-fat milk powder dissolved in Tris-buffered saline (TBS). Kunitz-PI;1 antiserum (Boex-Fontvieille et al., 2015a) was diluted 1:300 and the samples incubated at room temperature for 2 h. Excess antibodies were removed by consecutive washes in 0.05% (v/v) Tween 20 dissolved in TBS. In turn, incubation was carried out with secondary antibodies (goat anti-rabbit IgG conjugated with alkaline phosphatase, Sigma). After numerous washes, the antigen-antibody complexes were visualized with a solution containing 100 mM NaCl, 100 mM Tris-HCl, pH 9, 50 mM MgCl_2_, 0.5 mM NBT, and 0.5 mM BCIP. Slides were rinsed, mounted in water and viewed under a light microscope (Eclipse E-600, Nikon).

### cDNA Synthesis and Semi-quantitative PCR

Total RNA was isolated from etiolated seedlings and 2 μg were incubated in the presence of 300 pmols of oligo-dT primers at 65°C for 5 min. Subsequent synthesis of the first cDNA strand was carried out with reverse transcriptase at 42°C for 2 h as recommended by the supplier (SUPERSCRIPT II, Invitrogen). Reactions were stopped by incubation at 72°C for 10 min and a 1 μL aliquot (1/30 of the final reaction volume) was subjected to semi-quantitative PCR (Innis et al., 1990), using the following primer pairs: HEC1fow 5′-TAATGTGTTTGAAGGGTTCTG-3′; HEC1rev 5′-CCATATCGATCCCGAGGC-3′; NTTfow 5′-TTCTCATTGGC CCTACTCAG-3′; NTTrev 5′-TTGTTCTACCTCAGAGGCA GG-3′; ACTfow 5′-CAGAATCAGATATCTAAAAATCCCGGA AA-3′; ACTrev 5′-TGGGATGACATGGAGAAGAT-3′; Kunitz-PIfow 5′-TGGCATGAGGAAAAAGCCAAG-3′; Kunitz-PIrev 5′-TCAATGTTTTCTCAAGCTCAA-3′. PCR cycle numbers were 28 for *ACTIN*, 30 for *Kunitz-PI;1* and 32 each for *NTT* and *HECATE1*. Data are representative and refer to three independent biological experiments.

### Kunitz-PI;1 Promoter Activity Test

Plant tissues to be used to detect Kunitz-PI;1 promoter activity were first infiltrated in a solution containing 100 mM phosphate buffer, pH7, 10 mM EDTA, and 1 mM 5-bromo-4-chloro-3-indolyl-beta-D-glucuronic acid (X-Gluc, Sigma–Aldrich) for 10 min and then kept overnight at 37°C. Destaining was achieved by successively incubating plant tissue in solutions containing 50% (v/v), 75% (v/v), and 96% (v/v) ethanol. Finally, the tissues were transferred into a solution containing 50% (v/v) ethanol/30% (v/v) glycerol and viewed under a microscope (Eclipse E-600, Nikon) or binocular (SZX12, Olympus). Photos were taken with an Olympus DP70 camera.

### Whole-Plant Predation Assay

Whole-plant predation assays were carried out according to Farmer and Dubugnon (2009). Accordingly, *P. scaber* and *A. vulgare* were fed pesticide-free adult *Arabidopsis* WT plants, then starved for 3 days and transferred into a growth chambers filled with soil. Feeding activity was monitored with 1–3 arthrpods per liter soil for 4-days old WT, Kunitz-PI;1 knock-out mutant and Kunitz-PI;1 overexpressor seedlings as described (Boex-Fontvieille et al., 2015a). Populations of 120 seedlings were analyzed in three independent experiments and feeding scored by counting the number of plants with damaged apical hooks and/or dropped cotyledons. For diet feeding experiments, starved pillbugs and woodlice were transferred at high density (24 individuals) onto agar plates containing different concentrations of bacterially expressed, chemically purified Kunitz-PI;1 or commercial soybean trypsin inhibitor. Arthropod viability was assessed after 4–5 days and expressed as percentage of living to dead individuals.

## Results

### Activity of Bacterially Expressed *Arabidopsis* Kunitz-PI;1

In *Arabidopsis thaliana*, a small family of Kunitz-type protease inhibitors exists. The protein encoded by At1g72290 contains the Kunitz-motif [LIVM]-X-D-X-[EDNTY]-[DG]-[RKHDENQ] and a chlorophyll(ide)-binding motif related to one of the chlorophyll-binding motifs in LHCII, the major light-harvesting chlorophyll-binding proteins of photosystem II (Green and Kühlbrandt, 1995). Based on the presence of these motifs, this protein is related to the family of water-soluble chlorophyll proteins (WSCPs) of *Brassicaceae* (Satoh et al., 2001; Bektas et al., 2012; Boex-Fontvieille et al., 2015a,b). We renamed this protein to *A. thaliana* Kunitz-PI;1.

Gateway cloning was used to create constructs permitting *Kunitz-PI;1* expression in *E. coli* and subsequent protein purification and activity measurements (Boex-Fontvieille et al., 2015a,b; see also Materials and Methods). Two types of constructs were prepared, encoding the full-length protein and a variant lacking the predicted NH_2_-terminal, 23 amino acids comprising signal peptide for intracellular targeting^3^

**FIGURE 1:**
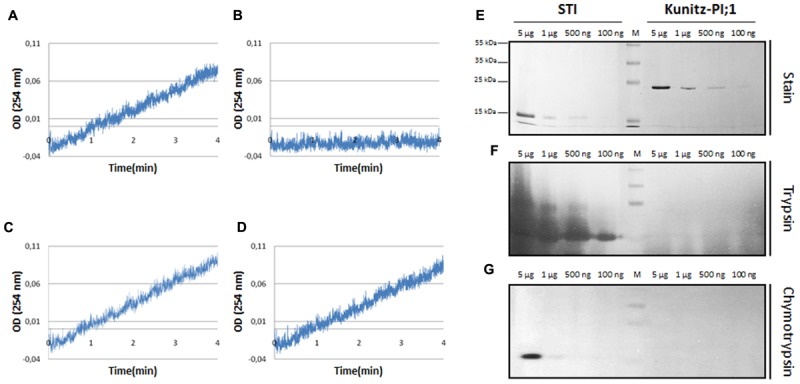
**Analysis of protease-inhibitor activity of bacterially expressed and purified *Arabidopsis* Kunitz-PI;1.** **(A–D)** Trypsin activity measured in the absence of protease inhibitors **(A)** or presence of either soybean trypsin inhibitor **(B)** or *Arabidopsis* Kunitz-PI;1 added at molar ratios of 1:1 **(C)** or 1:10 **(D)**. Trypsin activity was determined by cleavage of BAEE and measuring the absorption of the product at 253 nm. **(E–G)** Reverse zymography to reveal protease inhibitor activity of soybean trypsin inhibitor (STI, left part) and *Arabidopsis* Kunitz-PI;1 (right part). **(E)** PIs were separated on a SDS-PAA gel and stained with Coomassie brilliant blue. (**F** as **E**) But showing soybean trypsin inhibitor (STI) (left part) and *Arabidopsis* Kunitz-PI;1 (right part) on a SDS-PAA gel containing gelatine after renaturation and incubation with trypsin. (**G** as **F**) But depicting the results obtained with chymotrypsin.

Protease inhibitor tests were carried out with pancreas trypsin, a protease predicted to be a Kunitz-PI target, and papain. Pilot experiments revealed that Kunitz-PI;1 displayed an unusual target protease specificity. Instead of acting on trypsin, Kunitz-PI;1 inhibited papain (**Supplementary Figure S1**), a cysteine protease that had previously been identified as potential Kunitz-PI;1 target (Halls et al., 2006). This result was confirmed by additional activity measurements. Swine pancreas trypsin was incubated with benzoyl-L-arginine ethyl ester (BAEE) and cleavage of the substrate followed by absorbance measurements at 253 nm in assays containing or lacking *Arabidopsis* Kunitz-PI;1 added at a 1:1 or 1:10 molar ratio (**Figures 1C,D**). For comparison, parallel assays contained soybean trypsin inhibitor (**Figures 1A,B**).

Whereas trypsin activity was inhibited by added soybean trypsin inhibitor, *Arabidopsis* Kunitz-PI;1 was ineffective. This result is consistent with previous results (Boex-Fontvieille et al., 2015a,b) and was confirmed by reverse zymography. Soybean trypsin inhibitor and Kunitz-PI;1 were separated by SDS-PAGE on gels containing gelatine. After separation, the proteins were renatured and incubated in protease-containing solution which led to the digestion of gelatine and all of the other protein bands, except for the PI bands corresponding to soybean trypsin inhibitor (**Figures 1F,G**, respectively, left panels). By contrast, the Kunitz-PI;1 band was rapidly degraded, as demonstrated by Coomassie staining (**Figures 1F,G**, respectively, right panels). We concluded that *Arabidopsis* Kunitz-PI;1 is not active on serine proteases. In line with this view, Halls et al. (2006) showed that Kunitz-PI;1 inhibits cysteine proteases containing a granulin domain, such as papain, but has only weak or no activity toward serine and aspartate proteases. Another cysteine protease targeted by Kunitz-PI;1 is RESPONSIVE TO DESICCATION 21 (RD21; Boex-Fontvieille et al., 2015a,b).

### Expression Pattern of *A. thaliana* Kunitz-PI;1 *In planta*

Previously raised Kunitz-PI;1-specific antibodies (Boex-Fontvieille et al., 2015b) were used for carrying out expression and cytolocalization studies. These were combined with promoter activity measurements, using the *Kunitz-PI;1* regulatory region fused to the coding region of bacterial β-glucuronidase (GUS), and semi-quantitative reverse transcription (RT)-PCR analyses.

**FIGURE 2:**
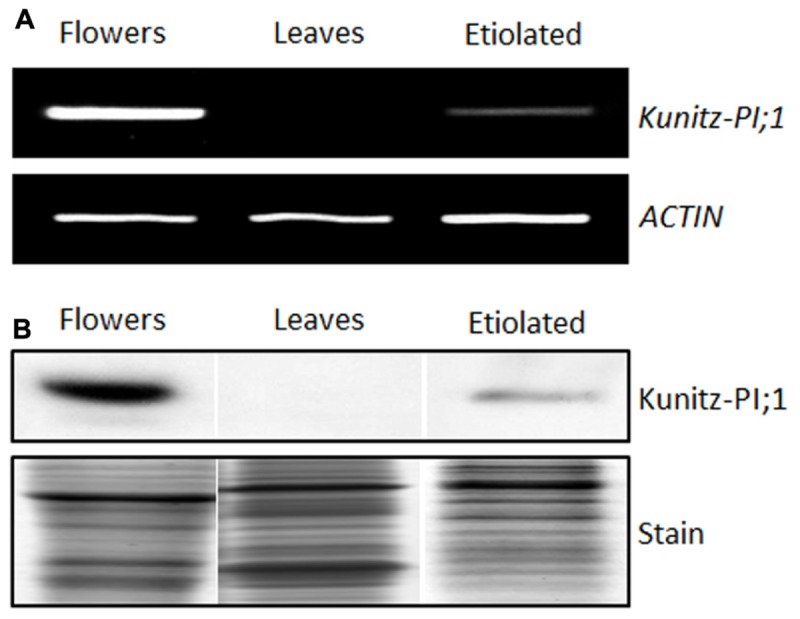
**Organ-specific expression of *Arabidopsis* Kunitz-PI;1.** **(A,B)** *Kunitz-PI;1* transcript **(A)** and Kunitz-PI;1 protein **(B)** abundance in flowers, in leaves of 3-week-old plants, and in 3-days old etiolated seedlings. The gel in **(A)** shows RT-PCR products visualized by ethidium bromide staining. Actin transcript levels were analyzed as internal constitutive marker. For SDS-PAGE **(B)**, 50 μg of total protein was loaded per lane. Replicate gels were used for immunodetection using Kunitz-PI;1-specific antibodies (upper panel) and Coomassie staining (lower panel). Note that **(B)** is a composite diagram.

According to information obtained from the Bio-Array Resource for Plant Functional Genomics^4^, Kunitz-PI;1 expression is high in dark-grown seedlings but low in light-grown seedlings (Winter et al., 2007). In mature plants, highest transcript levels were found in flowers (Winter et al., 2007). Confirming these results, *Kunitz-PI;1* RNA was detectable in etiolated seedlings and flowers. Semi-quantitative RT-PCR revealed that, compared to flower extracts, only small quantities of *Kunitz-PI;1* transcript accumulated in etiolated seedlings. No *Kunitz-PI;1* transcript was present in leaves of 3 weeks-old green plants (**Figure 2A**). Western blot analyses performed with the Kunitz-PI;1-specific antibody confirmed Kunitz-PI;1 protein accumulation in etiolated seedlings, although at much lower quantities than in flower extracts. Reflecting the absence of respective transcript, Kunitz-PI;1 protein was undetectable in leaves of 3 weeks-old, green plants and the cotyledons of 4-days old light-grown seedlings (**Figure 2B**).

### Transcription Factors NTT and HEC1 Regulate the Expression of Kunitz-PI;1 in Etiolated Seedlings

**FIGURE 3:**
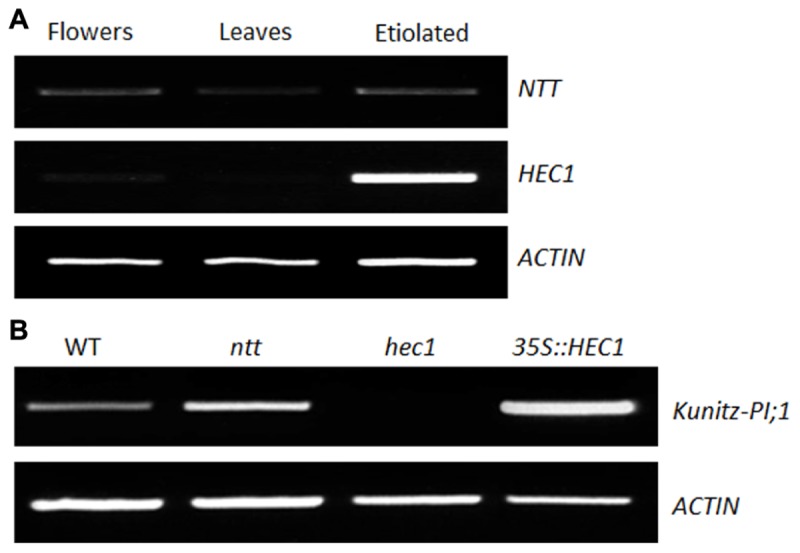
**Expression of *NTT* and *HEC1* genes in *Arabidopsis*.** **(A)** Semi-quantitative RT-PCR to analyze the expression of the *NTT* and *HEC1* genes in flowers, the leaves of 3-week-old plants, and in 3-days old etiolated seedlings. (**B** as **A**) But showing RT-PCR data for *Kunitz-PI;1* transcripts in *ntt* and *hec1* mutants as well as *35S::hec1* overexpressing lines after growth in darkness for 3 days. Actin transcript levels were assessed as internal standard.

No transmitting tract and HEC1 are transcription factors involved in female reproductive tract development in flowers of *Arabidopsis* (Crawford et al., 2007; Gremski et al., 2007). We asked whether NTT and HEC1 could regulate *Kunitz-PI;1* transcription also in etiolated seedlings. As a first step to answer this question, the presence of *NTT* and *HEC1* transcripts in etiolated seedlings was assessed by semi-quantitative RT-PCR. For comparison, RNA extracts from flowers and leaves were used as positive and negative controls, respectively. Indeed, *NTT* and *HEC1* transcripts were detectable in total RNA preparations of etiolated seedlings. Remarkably, *NTT* transcript abundance was comparable for flowers and etiolated seedlings (**Figure 3A**). *HEC1* transcripts, however, appeared at much higher abundance in etiolated seedlings than in flowers (**Figure 3A**). Only small amounts of *NTT* and *HEC1* transcript were present in leaves of adult plants (**Figure 3A**).

*ntt* and *hec1* knock-out mutants as well as a line overexpressing *HEC1* under control of the *35S*-cauliflower mosaic virus promoter (*35S::HEC1*) were obtained (Crawford et al., 2007; Gremski et al., 2007) and the presence of Kunitz-PI;1 transcript investigated by semi-quantitative RT-PCR. Accordingly, no *Kunitz-PI;1* transcript was detectable in the *hec1* mutant (**Figure 3B**), suggesting that HEC1 positively controls Kunitz-PI;1 accumulation. On the other hand, slightly higher levels of Kunitz-PI;1 transcript accumulated in the *ntt* mutant than in the WT (**Figure 3B**), indicating that NTT negatively regulates Kunitz-PI;1 expression in etiolated seedlings. Consistent with this view, higher levels of Kunitz-PI;1 transcript were detectable in *35S::HEC1* plants, as compared to WT plants (**Figure 3B**).

### Phytohormone Influence on the Accumulation of *Kunitz-PI;1* mRNA

Ethylene, brassinosteroids, gibberellins, jasmonic acid, and auxins regulate skotomorphogenesis and the switch to photomorphogenetic growth (see Introduction). We asked whether auxin (IAA) and ethylene could influence the expression of Kunitz-PI;1 in dark-grown *Arabidopsis* seedlings. Seeds of the *pKunitz-PI;1::GUS* line were germinated in the dark in the absence or presence of the ethylene precursor ACC or ethylene biosynthesis inhibitor silver nitrate. Parallel plates contained the auxin IAA, while mock plates lacked any additives. After 3 days in darkness, GUS activity was monitored.

**FIGURE 4:**
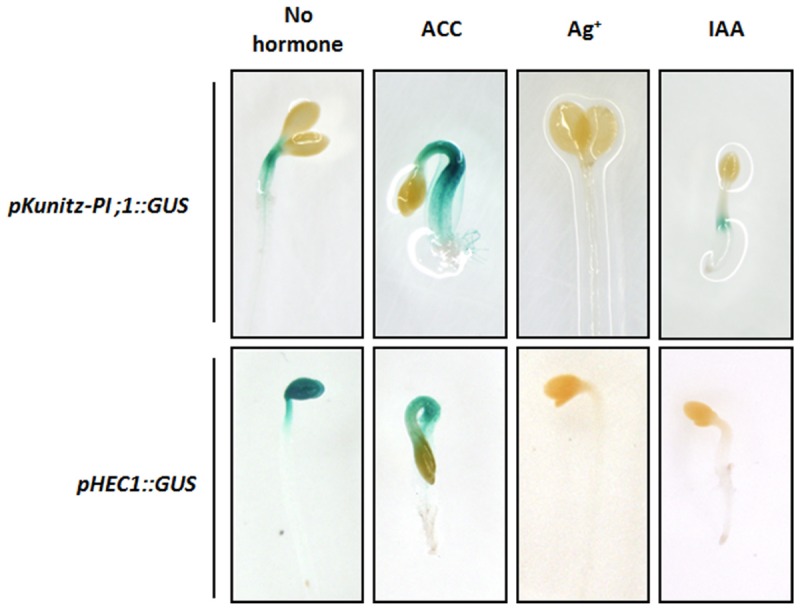
**Influence of phytohormones on *Kunitz-PI;1* promoter activity (**Upper** row) and *HEC1*-promoter activity (**Lower** row).** Etiolated seedlings of the *pKunitz-PI;1::GUS* and *pHEC1::GUS* lines, respectively, were grown on Murashige–Skoog medium containing ACC as ethylene precursor, silver nitrate as ethylene biosynthesis inhibitor, or IAA as auxin, for 3 days. Then, GUS activity was monitored to visualize promoter activity.

Confirming previous results (Boex-Fontvieille et al., 2015a), maximum *Kunitz-PI;1* promoter activity was detected in the apical hook of etiolated seedlings and especially in the vascular tissues (**Figure 4**). Etiolated seedlings that had been germinated in the presence of ACC exhibited the well-known ‘triple response,’ which comprises (i) inhibition of elongation and swelling of the hypocotyls, (ii) inhibition of root elongation, and (iii) exaggeration of apical hook curvature (Knight and Crocker, 1913). *Kunitz-PI;1* promoter activity seemed higher in the presence of ACC than in the hormone-free control and was located to the apical hook (**Figure 4**). When the ethylene biosynthesis inhibitor silver nitrate was present in the growth medium, *Kunitz-PI;1* promoter activity was abrogated (**Figure 4**). Similarly, IAA dropped *Kunitz-PI;1* promoter activity to negligible levels (**Figure 4**).

Etiolated seedlings that had been stably transformed with a fusion construct of the *HEC1* promoter and GUS (*pHEC1::GUS*) were next analyzed. In the absence of additives, *HEC1* promoter activity was easily detectable in the cotyledons and the apical hook. Exogenous application of ACC led to a change in the pattern of *HEC1* promoter activity that was reduced in the cotyledons but maintained in the hypocotyls (**Figure 4**). In the presence of either silver nitrate or IAA, *HEC1* promoter activity was completely abolished (**Figure 4**).

**FIGURE 5:**
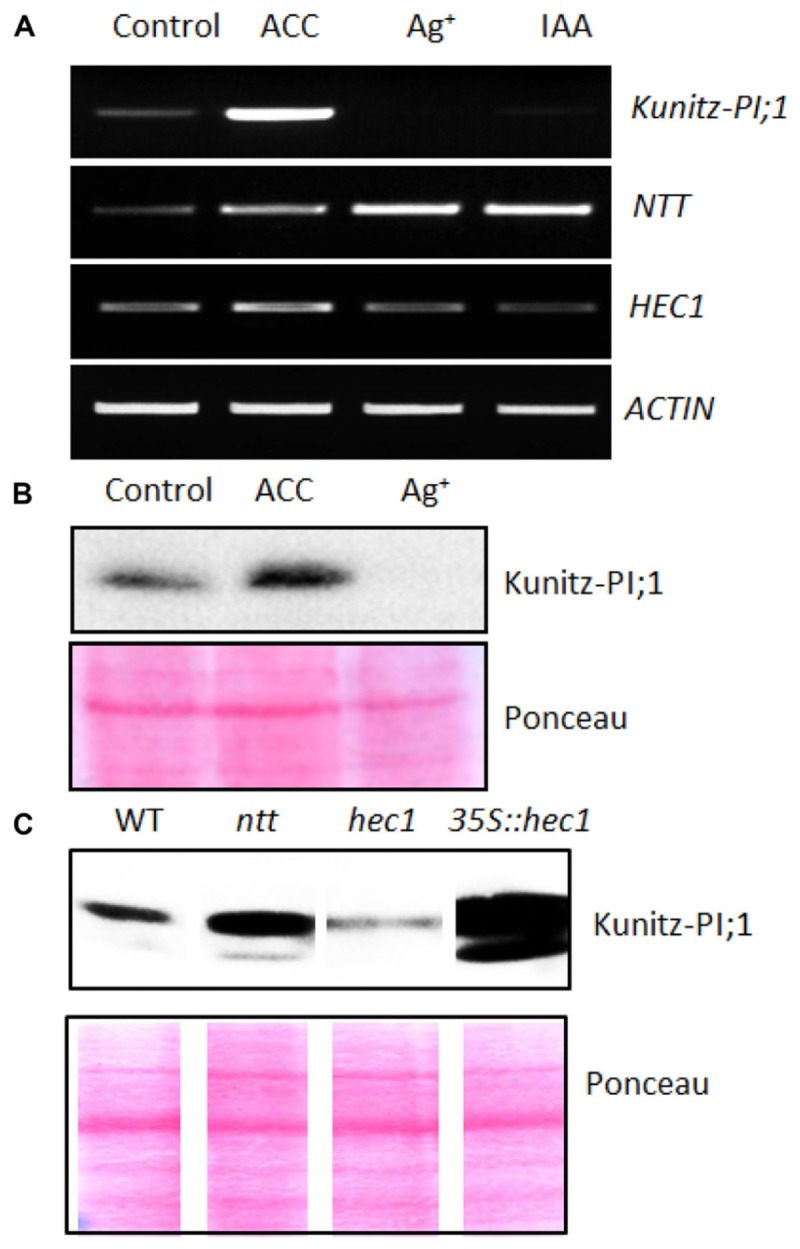
**Expression of *NTT* and *HEC1* versus *Kunity-PI;1* transcripts in response to phytohormones.** **(A)** Semi-quantitative RT-PCR analysis of *NTT*, *HEC1*, and *Kunitz-PI;1* expression in 3-days old etiolated seedlings grown on ACC-containing, silver nitrate-containing or IAA-containing Murashige–Skoog medium. For comparison, actin transcript levels were assessed as internal standard. **(B)** Kunitz-PI;1 protein levels in 3-days old etiolated seedlings after growth on ACC-containing or Ag^+^-containing medium analyzed by Western blotting. **(C)** Kunitz-PI;1 protein accumulation in etiolated seedlings of WT, *ntt* and *hec1* mutant, as well as *35S::hec1* overexpressor. For SDS-PAGE **(B,C)**, 40 μg protein was loaded per lane and subjected to Western blotting using Kunitz-PI;1-specific antibodies (upper panels); loading was confirmed by Ponceau-staining of the nitrocellulose-blotted proteins (lower panels). Note that the lower panels in **(C)** are composite diagrams.

Semi-quantitative RT-PCR was performed to correlate *Kunitz-PI;1* promoter activity with respective changes in transcript abundance. In addition to the *Kunitz-PI;1* transcript, *NTT*, *HEC1* and *ACTIN* transcripts were quantified by RT-PCR. **Figure 5A** shows that increased *Kunitz-PI;1* transcript levels were found in the presence of ACC, whereas no *Kunitz-PI;1* transcript was detectable in seedlings that had been grown on silver nitrate or IAA. Interestingly, similar effects were seen at the protein level (**Figure 5B**). Seedlings grown on ACC-containing medium expressed higher amounts of Kunitz-PI;1 protein than those grown on hormone-free medium. After growth in the presence of silver nitrate, Kunitz-PI;1 protein levels were negligibly low, in most cases undetectable (**Figure 5B**).

*NTT* transcript levels quantified in parallel were augmented in seedlings that had been treated with silver nitrate or IAA, as compared to seedlings grown on hormone-free medium (**Figure 5A**). By contrast, *HEC1* transcript levels were reduced on silver nitrate- and IAA-containing medium but increased in the presence of ACC (**Figure 5A**). In etiolated *ntt* mutant seedlings, more Kunitz-PI;1 protein was detected than in WT seedlings treated with ACC (**Figure 5C**). *hec1* mutant seedlings showed drastically reduced Kunitz-PI;1 protein levels in the presence of ACC. In line with this observation, the *HEC1* overexpressing line accumulated 10- to 20-fold more Kunitz-PI;1 protein in response to ACC than WT seedlings (**Figure 5C**).

### Immunolocalization of Kunitz-PI;1 Protein in Etiolated Seedlings after Ethylene Treatment

**FIGURE 6:**
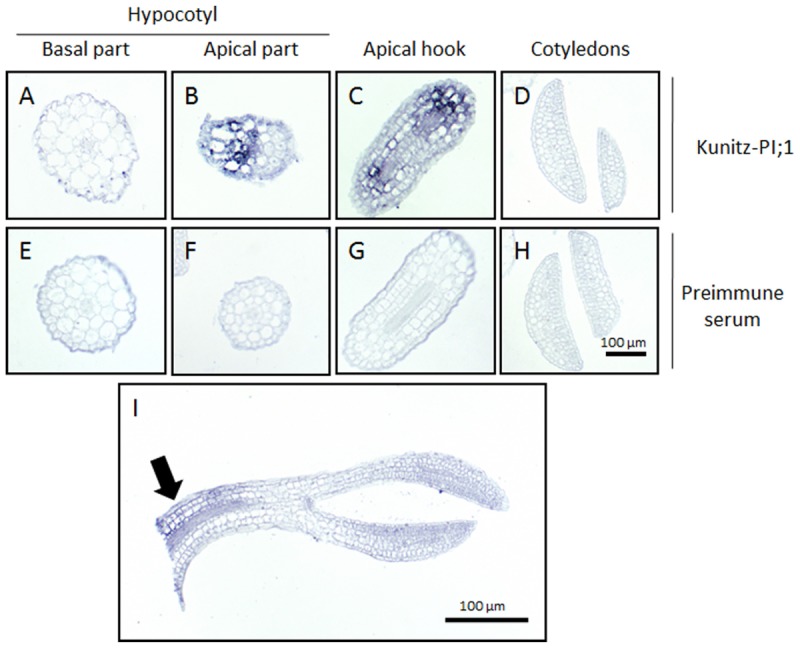
**Immunolocalization of *Arabidopsis* Kunitz-PI;1 protein.** Seedlings were grown for 3 days on ACC-containing medium. Tissue sections from the basal part of the hypocotyls **(A,E)** and apical part of the hypocotyls **(B,F)**, the apical hook **(C,G)**, the cotyledons **(D,H)**, and a longitudinal section of the upper half of a seedling **(I)** were incubated with Kunitz-PI;1-specific antibodies **(A–D,I)** or preimmune serum **(E–H)**. Detection of the antigen-antibody complexes was made using an alkaline phosphatase-based assay with NBT and BCIP.

The promoter-GUS studies had revealed strong Kunitz-PI;1 expression in the vascular tissues of the apical hook. To back-up this observation, the localization of Kunitz-PI;1 protein was assessed by carrying out *in situ*-localization studies. Whereas no Kunitz-PI;1 protein could be detected in the cotyledons (**Figure 6D**) and the basal part of the hypocotyls (**Figure 6A**), clear signals were obtained for the apical part of the hypocotyls (**Figure 6B**) and especially the apical hook region (**Figure 6C**). At the latter place, the protein was localized in the cortex and endodermis of the convex part (**Figures 6B,C,I**). No signal was obtained with a respective preimmune serum (**Figures 6E–H**).

### Induction of Kunitz-PI;1 Promoter Activity by Wounding

Ethylene functions as plant growth regulator at many stages of development and is also known to be a stress hormone mediating responses to mechanical constraints (Lin et al., 2009). Etiolated seedling need to protect their apical hooks and embedded meristem against mechanical damage when growing through the soil. We hypothesized that Kunitz-PI;1 could contribute to this protection mechanism and therefore tested *Kunitz-PI;1* promoter activity in response to wounding. 3-days old etiolated seedlings of the *pKunitz-PI;1::GUS* line were subjected to wounding, exerted by cutting the seedlings equidistantly from the apical hook and root tip or crushing the apical hook with tweezers. Afterward, the seedlings were kept in the dark for another 24 h-period before measuring *Kunitz-PI;1* promoter activity.

**FIGURE 7:**
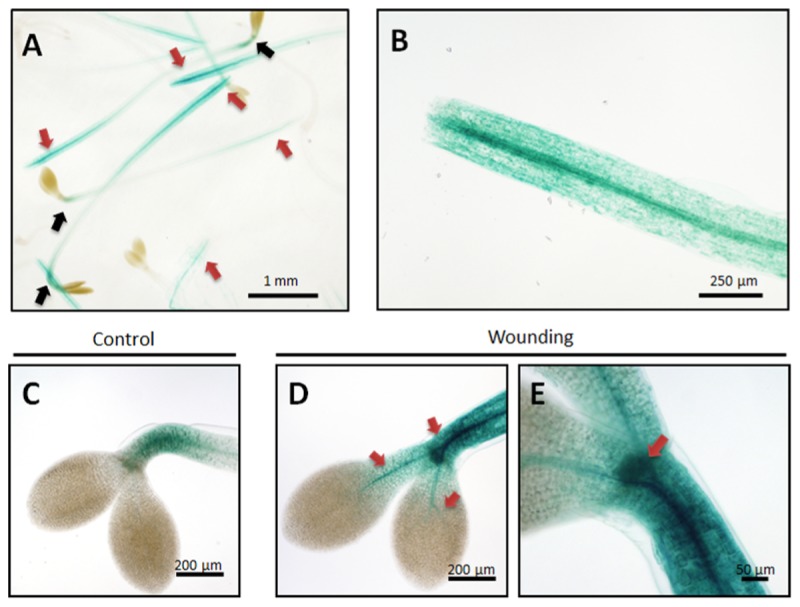
***Kunitz-PI;1* promoter activity in response to wounding.** **(A,B)** The hypocotyls of 3-days old etiolated seedlings were cut into two halves of which the aerial part comprising the apical hook and cotyledons **(A)** and the basal part comprising the root **(B)** were analyzed further by GUS staining. Red and black arrows mark regions of high and low *Kunitz-PI;1* promoter activity, respectively. **(C–E)** Etiolated seedlings were wounded by crushing the hypocotyls with tweezers near the apical hook region (red arrows) and GUS staining made 24 h thereafter **(D,E)**. **(C)** Unwounded control. Size markers are indicated.

**Figure 7** shows that cutting the seedlings into an upper, aerial part and lower, mostly root part, triggered local and systemic *Kunitz-PI;1* promoter activation in the cut hypocotyls but neither in the apical hooks nor cotyledons (**Figures 7A,B**, red versus black arrows). Crushing the apical hook led to a local up-regulation of *Kunitz-PI;1* promoter activity that now was no longer confined to the apical hook but was also detectable in the vascular bundles of the cotyledons (**Figures 7D,E**), as compared to the untreated controls (**Figure 7C**). Together, these results revealed that the *Kunitz-PI;1* promoter is wound-responsive.

### Ethylene Protects the Apical Hook against Arthropod Feeding

Ethylene-induced stem thickening and hook curvature are supposed to be measures against mechanical impedance (Harpham et al., 1991). Other reasons for the obvious phenotypic effects triggered by ethylene could be biotic foes such as arthropod isopods including pillbugs and woodlice that live underneath stones and fallen leaves and act as seed predators and facultative herbivores. We asked whether ethylene-triggered *Kunitz-PI;1* gene expression could contribute to the protection of etiolated seedlings against herbivorous arthropods such as *Porcellio scaber* (woodlouse) and *Armadillidium vulgare* (pillbug). Laboratory feeding experiments were carried out as described by Farmer and Dubugnon (2009). In addition to WT seedlings, a homozygous knock-out mutant line that contains a T-DNA insertion in the single exon of the *Kunitz-PI;1* gene was included (Boex-Fontvieille et al., 2015a). Furthermore, *Arabidopsis* WT plants that had been transformed with a construct containing the *Kunitz-PI;1* coding region under control of the *35S*-promoter (*35S::Kunitz-PI;1*; Boex-Fontvieille et al., 2015a) were used for comparison. These tools were employed to assess the role of Kunitz-PI;1 protein irrespective of the NTT and HEC1 transcription factors.

**FIGURE 8:**
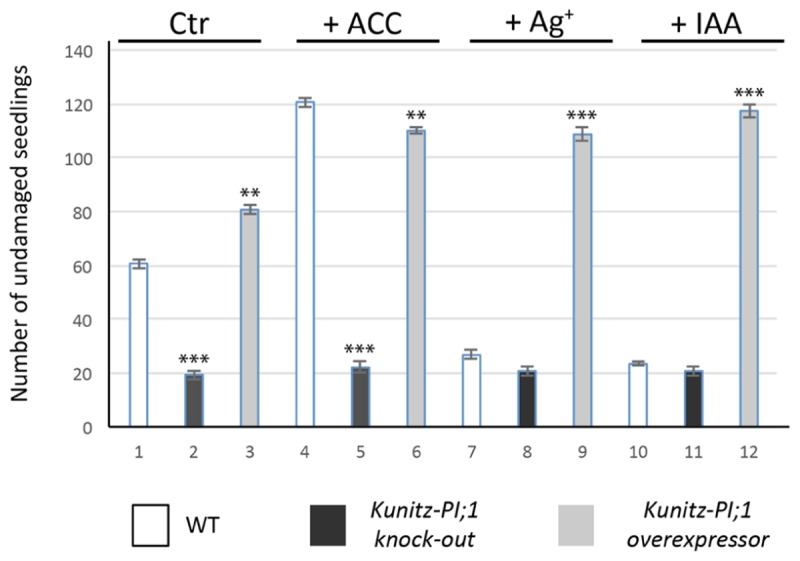
**Feeding of *P. scaber* on etiolated WT (white columns), *AtKunitz-PI;1* mutant (black bars) and *35S::Kunitz-PI;1* overexpressor (gray columns) seedlings.** In parallel assays, feeding was tested for seedlings that had been grown on agar plates containing the ethylene precursor ACC, ethylene biosynthesis inhibitor silver nitrate, or the auxin IAA. Mock plates (control, Ctr) lacked any additives. Isopod feeding activity was scored by counting the number of damaged versus undamaged seedlings and is expressed as number of undamaged seedlings in the population of 120 seedlings analyzed. Error bars refer to three independent experiments. Asterisks indicate statistically significant changes compared with the corresponding WT sample for each condition calculated by a two-tailed Student’s *t*-test; ^∗∗^*P* < 0.01, ^∗∗∗^*P* < 0.001 (cf. **Supplementary Table S1**).

Etiolated seedlings of the *Kunitz-PI;1* knock-out mutant as well as Kunitz-PI;1 overexpressing line showed no obvious phenotypic differences when grown on hormone-free medium, neither with regard to hypocotyl length nor apical hook structure (Boex-Fontvieille et al., 2015a) and all displayed the typical triple response when treated with ACC (**Supplementary Figure S2**). In laboratory feeding experiments with *Porcellio scaber* (woodlouse) and *Armadillidium vulgare* (pillbug), however, marked differences were observed. Ethylene caused a higher proportion of seedlings that were protected against pillbugs and woodlice feeding as compared to respective mock controls (**Figure 8**; see also **Supplementary Table S1** for a statistical analysis). This effect seemed to be, at least in part, due to the enhanced expression of Kunitz-PI;1. Supporting this view, seedlings of the *Kunitz-PI;1* knock-out mutant treated with ACC were much less protected against pillbug and woodlice attack than WT seedlings (**Figure 8**). The ethylene effect on WT seedlings was abrogated in the presence of silver nitrate as ethylene biosynthesis inhibitor or IAA. Overexpression of Kunitz-PI;1 gave rise to a seemingly phytohormone-insensitive, constitutive mechanism of arthropod deterrence of etiolated seedlings (**Figure 8**).

### Kunitz-PI;1 Efficiently Inhibits Cysteine Proteases of the Arthropod Gut

**FIGURE 9:**
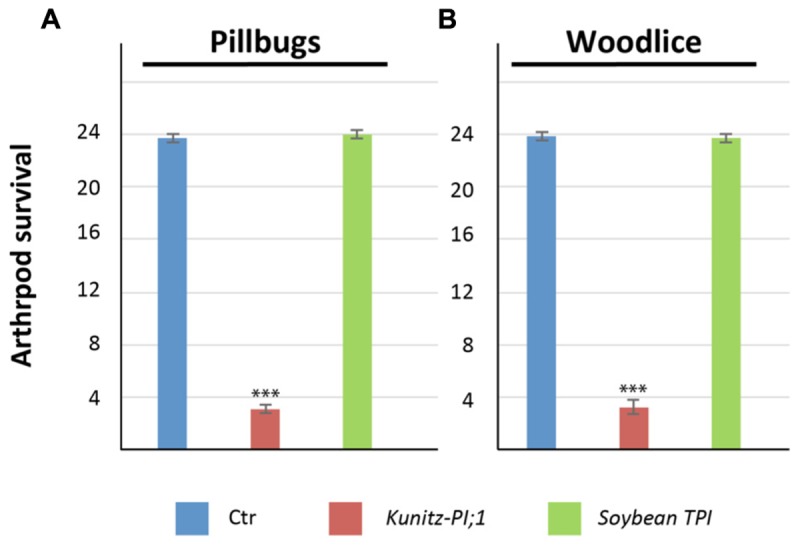
**Diet test conducted with bacterially expressed and purified *Arabidopsis* Kunitz-PI;1 and commercially available soybean trypsin inhibitor.** **(A)** Survival rates of nutrient-starved pillbugs on Kunitz-PI;1 and soybean trypsin inhibitor diets. For control (Ctr), a senescent *Arabidopsis* leave was used as diet. **(B)** as **(A)**, but depicting the results for nutrient-starved woodlice on Kunitz-PI;1 and soybean trypsin inhibitor versus control diets. Asterisks indicate statistically significant changes compared with the corresponding WT sample for each condition calculated by a two-tailed Student’s *t*-test; ^∗∗∗^*P* < 0.001 (cf. **Supplementary Table S2**).

The results presented thus far suggested that Kunitz-PI:1 may function in herbivore deterrence by inhibiting digestive proteases in the arthropod gut. To examine this point, laboratory feeding experiments were carried out with bacterially expressed and purified Kunitz-PI;1. As control, chemically pure soybean trypsin inhibitor was used. Both PIs were offered to pillbugs and woodlice that had been starved for nutrients for three days. **Figure 9** shows that Kunitz-PI;1 was a very poor diet for both arthropod species (see also **Supplementary Table S2** for a statistical analysis). By contrast, both arthropods rapidly consumed the soybean trypsin inhibitor diet. While pillbugs and woodlice easily survived if fed the soybean trypsin inhibitor, they passed away on Kunitz-PI;1 diet presumably as a result of nutrient deprivation and indigestibility of Kunitz-PI;1. Taking into account the known specificity of both PIs, we concluded that the arthropod gut of both crustacean is mostly containing cysteine proteases.

## Discussion

A member of the family of Kunitz-PIs in *A. thaliana* (encoded by At1g72290) was subject of this analysis. *Arabidopsis* Kunitz-PI;1, as we dubbed the protein here, has a molecular mass of ≈21 kDa which is similar to that of other Kunitz-PIs (Oliva and Sampaio, 2008, 2009). However, despite the presence of the family-defining Kunitz motif, *Arabidopsis* Kunitz-PI;1 was unable to inhibit trypsin and chymotrypsin (**Figure 1**). Although Kunitz-PIs mainly inhibit serine proteases, such as trypsin and chymotrypsin, some of them were shown to inhibit cysteine (thiol) or aspartate proteases (Rawlings et al., 2004). Even bifunctional activity, e.g., against both serine and cysteine proteases, has been reported (Franco et al., 2002). *Arabidopsis* Kunitz-PI;1 does not seem to exhibit bifunctional activity but was active on cysteine proteases possessing a granuline domain such as papain and RD21 (Halls et al., 2006; Boex-Fontvieille et al., 2015a,b).

Expression of *Arabidopsis* Kunitz-PI;1 was analyzed over plant development and in different plant organs. Kunitz-PI;1 transcript and protein accumulated in flowers and young siliques but not in leaves and roots of adult plants (**Figure 2**; cf. Boex-Fontvieille et al., 2015b). This result is in agreement with microarray data showing *Arabidopsis* Kunitz-PI;1 expression during flower development and especially in the female reproductive tract (Scutt et al., 2003; Tung et al., 2005; Bektas et al., 2012; Boex-Fontvieille et al., 2015b). Kunitz-PI;1 protein was not detected in dry seeds and thus is not a seed storage protein. Substantial *Kunitz-PI;1* transcript (**Figure 2A**) and Kunitz-PI;1 protein (**Figure 2B**) were detected in etiolated seedlings. Interestingly, the highest *Kunitz-PI;1* promoter activity was found in the region around the vascular bundles of the apical hook (**Figure 2C**). In chickpea, two Kunitz trypsin inhibitors were characterised, designated TPI-1 and TPI-2, of which TPI-1 accumulated in epicotyls and roots of etiolated seedlings (Jiménez et al., 2007, 2008; Hernández-Nistal et al., 2009). In either organ, TPI-1 was found in cell walls of vascular tissue and it was therefore proposed that TPI-1 could play a role in vascular tissue formation during growth. This function would be restricted to etiolated seedlings since TPI-1 expression was shown to be negatively light-regulated (Jiménez et al., 2007). We observed a similar, dark-specific expression for *Arabidopsis* Kunitz-PI;1 that was not detectable in greening seedlings and light-grown plants (**Figures 2A,B**). Moreover, *Arabidopsis* Kunitz-PI;1 protein was found in cell walls/apoplastic spaces by *in situ*-localization studies (Boex-Fontvieille et al., 2015a).

Considerable progress has been made during the last few years in the elucidation of the signal transduction cascades regulating skotomorphogenesis and photomorphogenesis. Generally, transcription factors that act as positive regulators of photomorphogenesis are degraded in the dark (Bae and Choi, 2008). On the other hand, transcription factors that negatively regulate photomorphogenesis, are degraded during greening (Kim et al., 2002). Here, we show that transcription factors such as NTT and HEC1 that regulate female reproductive tract development in *Arabidopsis* flowers (Crawford et al., 2007; Gremski et al., 2007) also are operative during skotomorphogenesis (**Figures 2A** and **3A**). While HEC1 functioned as positive regulator of *Kunitz-PI;1* gene expression, NTT acted negatively (**Figures 2B** and **3B**). How HEC1 and NTT may accomplish their roles in dark-grown seedlings and how they interact with other known components of the PHY-mediated signal transduction chain needs to be resolved in future work. Zhu et al. (2016) provided evidence for a negative feedback loop between key players of phytochrome signal transduction, such as the phytochrome-interacting factors, and HECATE proteins.

HECATE1 and NTT appear to be part of a phytohormone response pathway that controls *Kunitz-PI;1* gene expression during skotomorphogenesis and greening (**Figure 5A**). In the presence of IAA, expression of the *Kunitz-PI;1* gene was abrogated through a mechanism involving NTT and HEC1 that were up and down-regulated, respectively. The proposed positive role of HEC1 on transcription of the *Kunitz-PI;1* gene was strengthened by co-localization of *HEC1* and *Kunitz-PI;1* promoter activities in the apical hook (**Figure 4**).

Ethylene (produced from its precursor ACC in the medium) caused an increase of *Kunitz-PI;1* promoter activity around the vascular bundles of the apical hook (**Figure 4**), which resulted in higher transcript (**Figure 5A**) and protein levels (**Figure 5B**) in etiolated seedlings. Furthermore, Kunitz-PI;1 protein accumulation in the cortex and endodermis of the convex part of the apical hook (**Figure 6**) was dependent on HEC1 and NTT (**Figure 5C**). When ethylene biosynthesis was blocked by the application of silver nitrate, Kunitz-PI;1 promoter activity was undetectable (**Figure 4**). Based on these results we conclude that the expression of *Arabidopsis* Kunitz-PI;1 is normally induced by endogenous ethylene produced in the apical hook region. As shown previously, ethylene production is high in the apical hook of dark-grown seedlings of different plant species such as pea (Goeschl et al., 1967) and bean (Kang et al., 1967). Both, ethylene production and hook formation were negatively regulated by red light in dark-grown bean seedlings (Kang et al., 1967).

In *Arabidopsis*, a change in the sensitivity to ethylene in response to red, far red or blue light was reported to be responsible for hook opening (Knee et al., 2000). The fact that Kunitz-PI;1 expression was highest in the apical hook and further up-regulated by exogenous ethylene suggests a direct involvement of this PI in hook formation and/or maintenance. However, when etiolated seedlings of WT, Kunitz-PI;1 knock-out mutant and Kunitz-PI;1 overexpressing lines were compared, no difference in either apical hook formation and structure or hypocotyl growth was observed (**Supplementary Figure S2**), disproving the idea of Kunitz-PI;1 operating as structural component. On the other hand, no difference in their physiological responses to the exogenous addition of phytohormones became apparent such as the well-known triple response to ethylene (**Supplementary Figure S2**). Illumination of etiolated seedlings showed that all three genotypes were unimpaired in hook opening, cotyledon expansion and greening (Boex-Fontvieille et al., 2015a).

A clue toward the understanding of Kunitz-PI;1’s physiological function was provided by the pioneering work of Harpham et al. (1991) who suggested a role of ethylene under conditions of mechanical impedance. Through its effect on stem thickening and hook curvature, ethylene was proposed to permit unhindered seedling growth through the soil during skotomorphogenesis. We were able to demonstrate that *Arabidopsis Kunitz-PI;1* promoter activity was induced after wounding (**Figure 7**) and that this response may be part of a defense mechanism against herbivorous arthropods, living underneath stones and fallen leaves, such as pillbugs and woodlice. These nocturnal isopods are detritivores but can also live as facultative herbivores that feed on etiolated seedlings. Pillbugs and woodlice are seed predators but also ingest the apical hook and drop the cotyledons that are later consumed as main nutrient source (Boex-Fontvieille et al., 2015a). The apical hook provides the Achilles’ heel of etiolated seedlings that is protected, as we show here (**Figure 8**), by an ethylene-mediated mechanism involving Kunitz-PI;1 and maybe other PIs. It is attractive to suppose that Kunitz-PI;1 operates as inhibitor of digestive proteases of the arthropod gut when these begin their feeding on etiolated seedlings. Laboratory feeding experiments revealed that Kunitz-PI;1 is a poor diet for nutrient-deprived pillbugs and woodlice, indicating the presence of cysteine proteases that were rendered inactive. In other systems, both serine and cysteine PIs have been identified as main digestive proteases. For example, *Nicotiana attenuata* plants attacked by herbivore such as *Manduca sexta* accumulate trypsin PIs (Zavala et al., 2004), the neurotoxin nicotine (Baldwin, 1999), and various secondary metabolites comprising oxylipins (Wu et al., 2008; see Howe and Jander, 2008; Wu and Baldwin, 2010, for reviews). Serine proteases have been detected in many insect orders, including *Lepidoptera*, *Diptera*, *Orthoptera*, *Hymenoptera*, and *Coleoptera* (Christeller et al., 1992). Analysis of larval midgut extracts of 12 lepidopteran species revealed that most of them use a trypsin- and elastase-based digestive system, whereas others depend on chymotrypsins (Christeller et al., 1992). Both trypsin and chymotrypsin-like enzymes have been identified in the tobacco budworm (*Heliothis virescens*; Johnston et al., 1995). Cysteine protease activity has been detected in many coleopterans, such as corn rootworm (*Diabrotica virgifera*; Koiwa et al., 2000), Colorado potato beetle (*Leptinotarsa decemlineata*; Gruden et al., 2003), and sugarcane weevil (*Sphenophorus levis*; Soares-Costa et al., 2011). In summary, the complement of digestive proteases in phytophagous insects is quite complex and comprises all catalytic types, i.e., serine, threonine, cysteine, aspartic, and metallo enzymes (Terra and Ferreira, 1994). Their activity is counteracted by plant PIs as part of their natural plant defense against herbivores (e.g., Bode et al., 2013; see Hartl et al., 2011; Zhu-Salzman and Zeng, 2015, for reviews).

The wound set by arthropod feeding in the model system studied here, obviously elicits accumulation of ethylene, leading to a boost in Kunitz-PI:1 expression and maybe the induction of other direct and indirect defenses aimed to prevent mass ingestion of larger seedling populations. Such direct and indirect defenses would comprise hypocotyl thickening and exaggerating apical hook curvature (Knight and Crocker, 1913). A subsequent, second line of defense could be the release of the defense protein RD21 to degrade via its intrinsic activity as cysteine protease digestive proteases of the arthropod gut during the subtle period when etiolated seedlings break through the soil and need to open the apical hook for cotyledon expansion and greening, permitting the switch to photomorphogenetic growth (Boex-Fontvieille et al., 2015a). This hypothetical model would not exclude the operation of additional mechanisms. For example, an unusual serine protease was described for *Arabidopsis* and to function in resistance to both necrotrophic fungi and insect herbivory (Laluk and Mengiste, 2011). Work is needed to identify the network of ethylene-response genes operating in the apical hook of etiolated seedlings and define their functions in induced arthropod deterrence.

## Author Contributions

EB-F: Carried out research and analyzed data. SRu: Carried out research and analyzed data. DvW: Analyzed data. SP: Carried out research, analyzed data. CR: Designed research, analyzed data, and wrote the paper. SRe: Designed research, carried out research, analyzed data, and wrote the paper.

## Conflict of Interest Statement

The authors declare that the research was conducted in the absence of any commercial or financial relationships that could be construed as a potential conflict of interest.

## Footnotes

1 www.cbs.dtu.dk/services/TargetP

2 http://www.ihcworld.com/_protocols/epitope_retrieval/citrate_buffer.htm.

3 http://www.cbs.dtu.dk/services/TargetP/.

4 www.bar.utoronto.ca

## Abbreviations

ACC: 1-aminocyclopropane-1-carboxylic acid
HEC1: HECATE 1
HR: hypersensitive response
IAA: indole acetic acid
NTT: no transmitting tract
PCD: programmed cell death
PI: protease inhibitor
