# Corrigendum: An Ethylene-Protected Achilles' Heel of Etiolated Seedlings for Arthropod Deterrence

**DOI:** 10.3389/fpls.2018.01741

**Published:** 2018-12-07

**Authors:** Edouard Boex-Fontvieille, Sachin Rustgi, Diter von Wettstein, Stephan Pollmann, Steffen Reinbothe, Christiane Reinbothe

**Affiliations:** ^1^Laboratoire de Génétique Moléculaire des Plantes and Biologie Environnementale et Systémique, Université Grenoble-Alpes – Laboratoire de Bioénergétique Fondamentale et Appliquée, Grenoble, France; ^2^Department of Agricultural and Environmental Sciences–Pee Dee Research and Education Center, Clemson University, Florence, SC, United States; ^3^Department of Crop and Soil Sciences – Center for Reproductive Biology, School of Molecular Biosciences, Washington State University, Pullman, WA, United States; ^4^Centro de Biotecnología y Genómica de Plantas, Univerdidad Politécnica de Madrid – Instituto Nacional de Investigación y Tecnología Agraria y Alimentación, Madrid, Spain

**Keywords:** skotomorphogenesis, apical hook, *Arabidopsis thaliana*, protease inhibitor action, herbivore deterrence

In the original article, there was a mistake in Figure 5 Expression of NTT and HEC1 versus Kunity-PI;1 transcripts in response to phytohormones, as published. Due to a technical error, the same image was shown twice in the lowermost panel of Figure 5C (Ponceau stains) for the *hec1* and *35S::hec1* lines. The original Ponceau stain as well as corrected Figure 5 Expression of NTT and HEC1 versus Kunity-PI;1 transcripts in response to phytohormones, appears below. The authors apologize for this error and state that this does not change the scientific conclusions of the article in any way. The original article has been updated.

**Figure 5:**
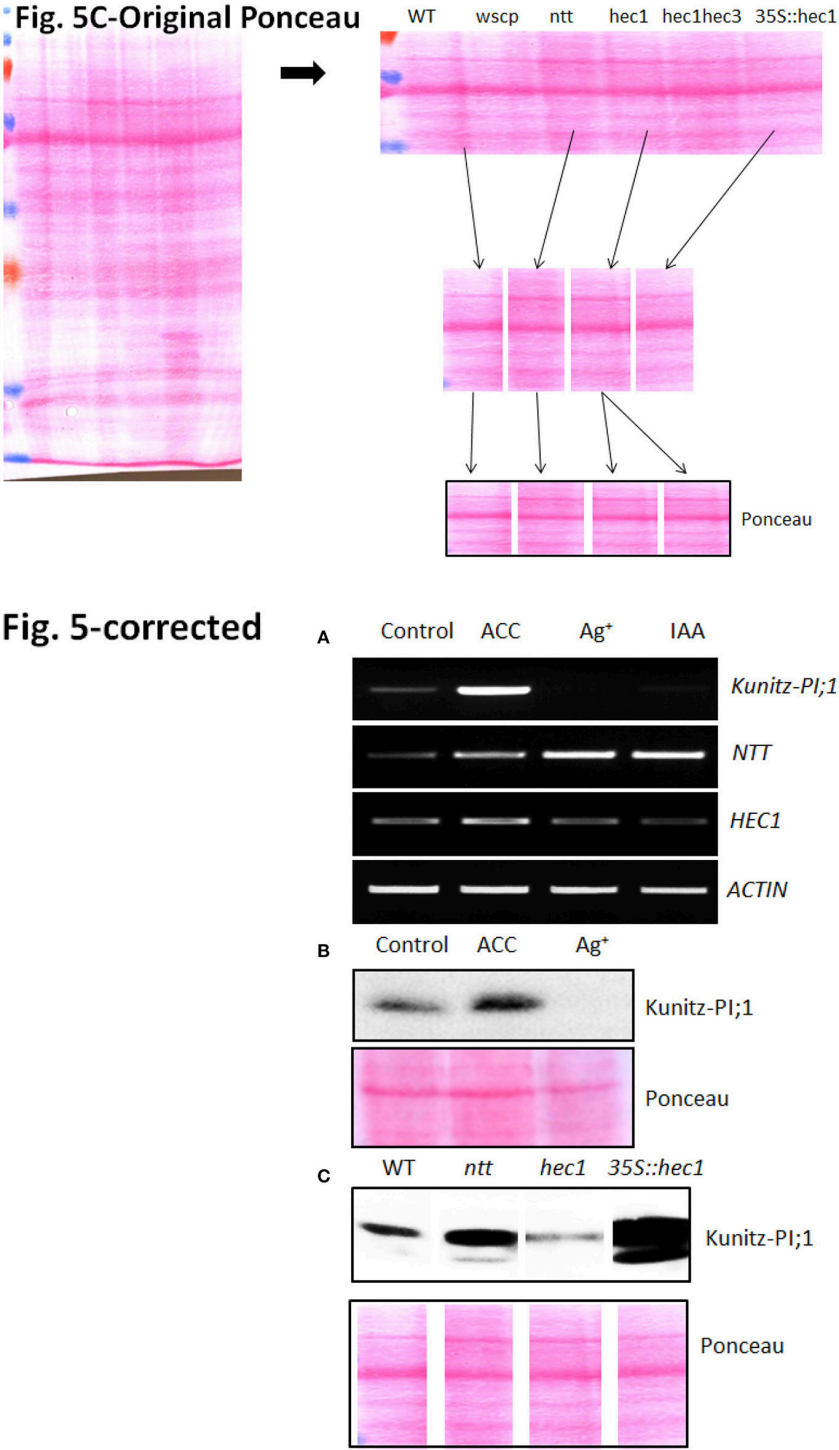
Expression of *NTT* and *HEC1* versus *Kunity-PI;1* transcripts in response to phytohormones. **(A)** Semi-quantitative RT-PCR analysis of *NTT, HEC1*, and *Kunitz-PI;1* expression in 3-days old etiolated seedlings grown on ACC-containing, silver nitrate-containing or IAA-containing Murashige–Skoog medium. For comparison, actin transcript levels were assessed as internal standard. **(B)** Kunitz-PI;1 protein levels in 3-days old etiolated seedlings after growth on ACC-containing or Ag^+^-containing medium analyzed by Western blotting. **(C)** Kunitz-PI;1 protein accumulation in etiolated seedlings of WT, *ntt* and *hec1* mutant, as well as *35S::hec1* overexpressor. For SDS-PAGE **(B,C)**, 40 μg protein was loaded per lane and subjected to Western blotting using Kunitz-PI;1-specific antibodies (upper panels); loading was confirmed by Ponceau-staining of the nitrocellulose-blotted proteins (lower panels). Note that the lower panels in **(C)** are composite diagrams.

## Conflict of interest statement

The authors declare that the research was conducted in the absence of any commercial or financial relationships that could be construed as a potential conflict of interest.

