# Supplementary material for: An Ethylene-Protected Achilles’ Heel of Etiolated Seedlings for Arthropod Deterrence
Source: Front Plant Sci. 2016 Aug 30;7:1246. doi: 10.3389/fpls.2016.01246 (PMC5003848; doi:10.3389/fpls.2016.01246)
Supplement: Supplementary file 3 [file Presentation_1.PDF]

Supplementary materials

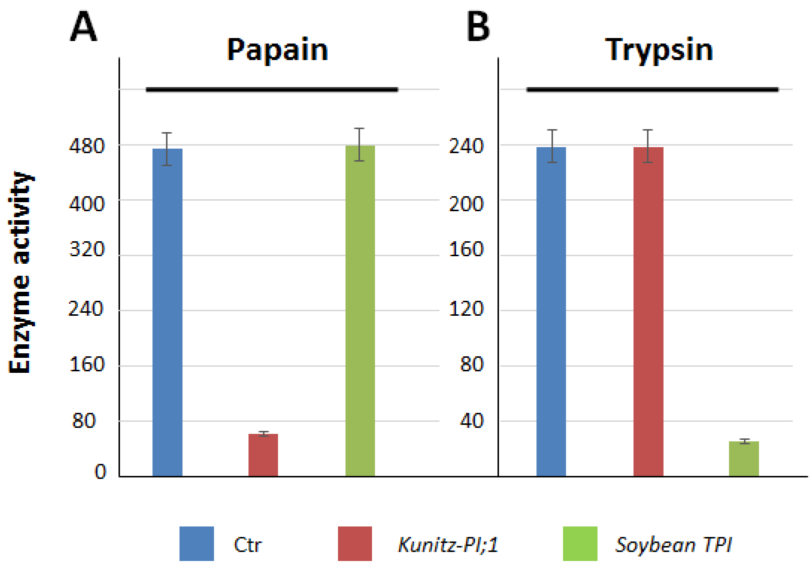

Fig. S1 Protease inhibitor test to define the specificity of bacterially expressed and purified Arabidopsis Kunitz-PI;1. **A,** Inhibition of papain, a cysteine protease, by Kunitz-PI;1. For comparison, enzymatic tests were carried out with soybean trypsin inhibitor (TPI) but failed to inhibit papain. Respective mock incubations (Ctr, control) lacked any protease inhibitor. **B,** as A, but showing activity measurements carried out with trypsin, a serine protease. Note the inhibition of trypsin by soybean trypsin inhibitor (TPI) but not by Kunizt-PI;1.

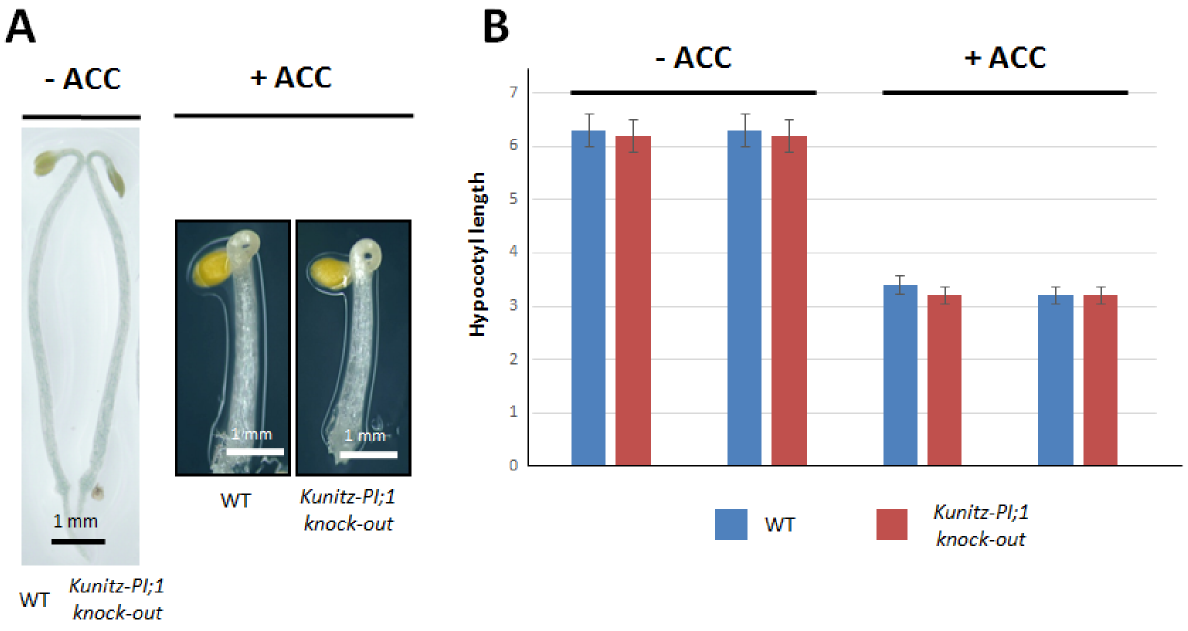

Fig. S2 A, Phenotype of 3 d-old etiolated wild-type (WT) and *Kunitz-PI;1* knock-out (KO) mutant seedlings in the absence (-) or presence (+) of ACC as ethylene precursor. Seeds were germinated on Murashige-Skoog medium containing 10 µM ACC and seedling morphologies compared. Size bars are indicated. **B,** Hypocotyl length of wild-type (WT) and *Kunitz-PI;1* knock-out (KO) mutant seedlings after growth on ACC-free and ACC-containing medium. The data represent the mean of three independent replicates comprising 40 seedlings each. Error bars are indicated.
